# Exosomes and Immune Response in Cancer: Friends or Foes?

**DOI:** 10.3389/fimmu.2018.00730

**Published:** 2018-04-11

**Authors:** Francisco M. Barros, Fatima Carneiro, Jose C. Machado, Sónia A. Melo

**Affiliations:** ^1^Faculty of Medicine of the University of Porto (FMUP), Porto, Portugal; ^2^Department of Pathology, Centro Hospitalar de São João, Porto, Portugal; ^3^Department of Pathology, Faculty of Medicine of the University of Porto (FMUP), Porto, Portugal; ^4^Institute for Research Innovation in Health (i3S), Porto, Portugal; ^5^Institute of Molecular Pathology and Immunology of the University of Porto (Ipatimup), Porto, Portugal

**Keywords:** exosomes, cancer, immune response, extracellular vesicles, clinical trials as topic

## Abstract

Exosomes are a type of extracellular vesicle whose study has grown exponentially in recent years. This led to the understanding that these structures, far from being inert waste by-products of cellular functioning, are active players in intercellular communication mechanisms, including in the interactions between cancer cells and the immune system. The deep comprehension of the crosstalk between tumors and the immune systems of their hosts has gained more and more importance, as immunotherapeutic techniques have emerged as viable options for several types of cancer. In this review, we present a comprehensive, updated, and elucidative review of the current knowledge on the functions played by the exosomes in this crosstalk. The roles of these vesicles in tumor antigen presentation, immune activation, and immunosuppression are approached as the relevant interactions between exosomes and the complement system. The last section of this review is reserved for the exploration of the results from the first phase I to II clinical trials of exosomes-based cell-free cancer vaccines.

## Introduction

The understanding of the intercellular communication processes is a key for the development of mechanistic insights capable of explaining a wide variety of both physiological and pathological phenomena. Direct cell-to-cell contact, and paracrine and endocrine interactions are relatively well-understood mechanisms that can account for some of these processes. However, a novel mechanism has emerged, involving the intercellular transfer of molecular and genetic material through extracellular vesicles (EVs), gaining considerable attention in recent years (1).

Extracellular vesicles are small phospholipid bilayer vesicles, released by all prokaryotic and eukaryotic cells, including cancer cells (2–5), which can contain different types of RNA, proteins, mitochondrial DNA, and both single stranded DNA and double stranded DNA, spanning all chromosomes (3, 6–8). The nomenclature of EVs has been a source of confusion due to the difficulties posed by the purification methods necessary for the distinction of the various types of EVs, and a definitive classification system has not been achieved yet (5, 9). However, EVs can be broadly classified according to their size and mode of biogenesis into three subtypes (1): microvesicles (MV), ranging between 50 and 1,000 nm in diameter, and originating by budding from plasma membranes (2). Apoptotic bodies (AB), which are 50–5,000 nm in diameter and originate from cells undergoing programmed cell death (3). Last, exosomes range between 30 and 150 nm in diameter, and originate from early endosomes, which are later transformed into multivesicular bodies (MVB) by formation of intraluminal vesicles (ILV) by budding into the lumen, followed by fusion with the plasma membrane and release of the ILV into the extracellular space as exosomes (2, 3, 5, 10). It is worth noting that cancer cells tend to release more exosomes than healthy cells, which may be due to enhanced growth rate or a result of stimulation in response to stressful conditions (11).

**Figure 1:**
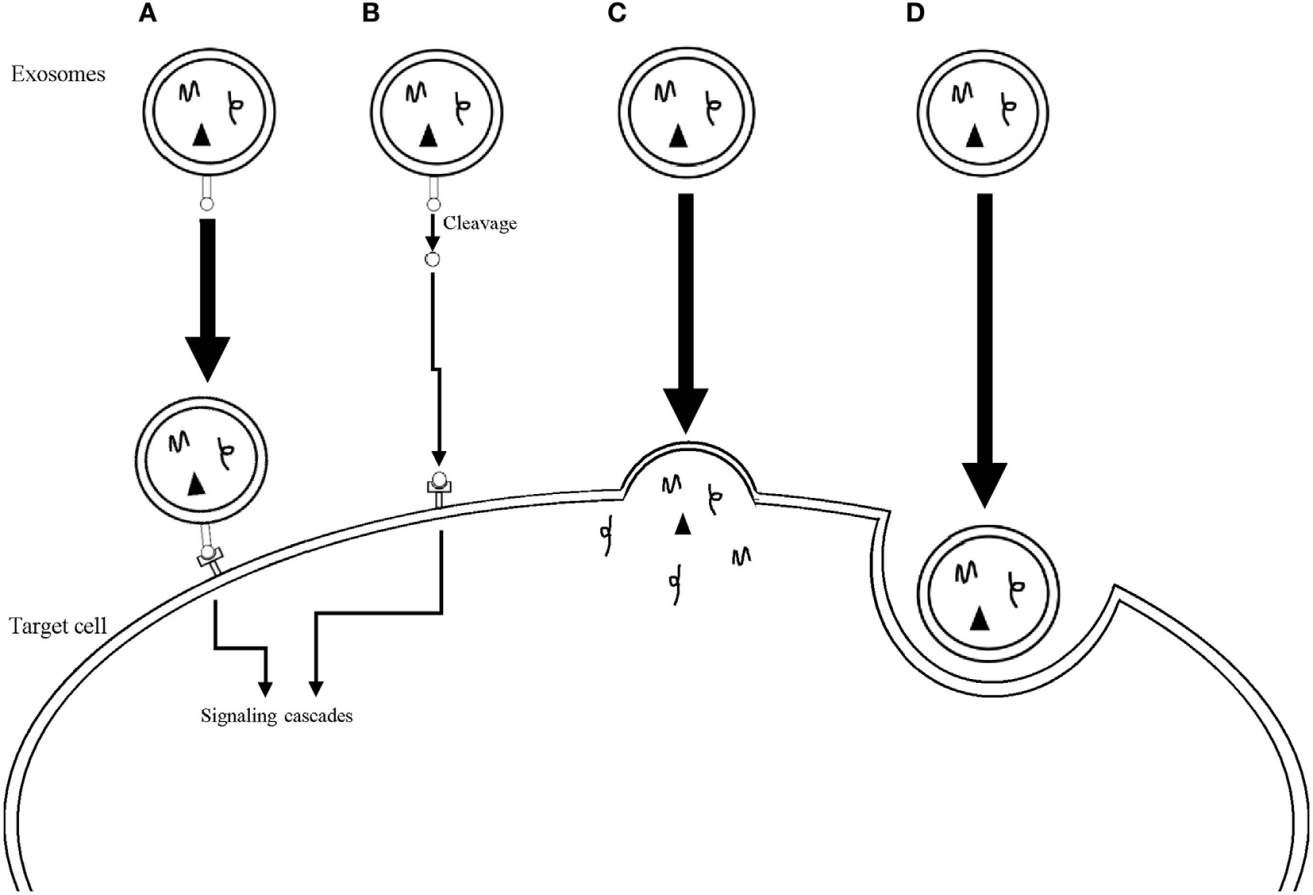
Mechanisms used by exosomes to influence target cells. **(A)** Direct interaction between surface receptors on the exosome and on the target cell. **(B)** Cleavage of surface receptors on the exosome with subsequent interaction between the receptor fragments and receptors on the target cell. **(C)** Fusion of the exosome membrane with the plasma membrane of the target cell with release of the exosomal cargo into the cytoplasm of the cell. **(D)** Internalization of the whole exosome through phagocytosis.

Exosomes have been isolated from all biological fluids tested so far, such as urine, breast milk, plasma, saliva, cerebral spinal fluid, amniotic fluid, ascites, bile, semen, bronchoalveolar lavage fluid, and aqueous humor (12–24). Exosomes contain collections of proteins, some of which show specificity for the cell type that originated them, such as MHC class I and II proteins, while others are present in all exosomes, regardless of the cell originating them, suggesting that the latter are related to the common biogenesis pathway of these EVs. Indeed, this group includes endosomal proteins, proteins from the plasma membrane and from the cytosol. Also, as a consequence of its genesis, proteins on the surface of exosomes have the same orientation as the one in their cell of origin (2, 5, 25). Exosomes are also packed with different types of nucleic acids, including DNA in some cases (6, 8, 26), but mostly small RNAs, such as ribosomal RNA, transfer RNA, miRNA (microRNAs), and messenger RNA (mRNA), that are selectively loaded into the vesicles. The mechanisms behind the enrichment in their cargo are still far from fully understood, but the presence of “zipcode” sequence motifs and posttranscriptional changes are some of the ways in which specific mRNAs and miRNAs can be packaged into exosomes (26). Exosomes can influence target cells through at least four different mechanisms (Figure 1) (1): direct contact between proteins on the membrane of exosomes and on the plasma membrane of the recipient cell, with subsequent triggering of intracellular signaling cascades (2). Cleavage of the proteins on the exosomes membrane followed by interaction between the protein fragments and membrane receptors on the cell (3). Fusion of the exosomes with the membrane of the cell leading to release of its cargo (4). Finally, cellular internalization of the whole vesicle by phagocytosis is also possible (2, 27, 28).

Immune cells of both the adaptive and innate systems are an important component of the tumor microenvironment that most times presents paradoxical roles in tumorigenesis. On the one hand, chronic inflammatory states can serve as agents for cancer initiation and promotion and can stimulate angiogenesis and metastasis (29). On the other hand, the immune system is also responsible for the specific identification and elimination of neoplastic cells. This process, known as cancer immunosurveillance, is based on the expression of tumor-specific antigens (30–33). The concept of immunosurveillance has been updated according to clinical and experimental data to include the notion that the immune system is not only involved in anti-tumor activity, but also shapes the tumor itself, resulting in the formulation of the cancer immunoediting hypothesis. This hypothesis views the interactions between the immune system and neoplasms as a continuum consisting of at least three components: (1) elimination, roughly corresponding to the concept of immunosurveillance. (2) Equilibrium, a state where net tumor cell outgrowth is kept in check by the immune cells, and there is no clinically apparent disease. (3) Last, there is escape, when cancer cells grow immunologically unrestricted due to Darwinian selection processes of the cells most fit to evade the immune control (33). Exosomes have recently emerged as important modulators of the immune response in the context of cancer development. These EVs can regulate the formation of the immunological synapse between T-cells and antigen-presenting cells (APC), promote the development of an immune response, and tumor-derived exosomes are part of the immunosuppressive mechanisms through which cancer cells inhibit immunosurveillance processes in order to progress and invade (25, 34–36).

This review will cover the current body of evidence regarding the roles of exosomes in these biological processes, as well as summarize their potential translational applications, both in therapeutic and diagnostic procedures. Conclusions from first phase I and II clinical trials of exosomes-based cell-free cancer vaccines are also reviewed, and the interactions between exosomes and the complement system will be briefly approached. We hope to present a clear, updated, and comprehensive insight into this rapidly evolving subject.

## Exosomes and Antigen Presentation

Antigen presentation constitutes a fundamental step of the immune response. This is the process through which APC, such as dendritic cells (DC), macrophages and B-cells, expose peptide antigens, bound to MHC class I or class II molecules, to T-cells by forming a contact point between the two cells, termed an immunological synapse (34). MHC class II molecules, specific to APC, are involved in the activation of CD4^+^ helper T-cells through the presentation of exogenous peptides, internalized by endocytosis. MHC class I molecules, present in all nucleated cells, are necessary for the widespread surveillance of the health status by CD8^+^ cytotoxic T lymphocytes (CTL) and natural killer (NK) cells (37). They are also involved in the contacts established between APC and CTL, and the peptides presented by MHC class I molecules are mostly of an endogenous origin, being generated by the proteasome. However, the presentation of exogenous antigens in complexes with MHC class I is also possible through cross-presentation, a process that is likely necessary for the establishment of an anti-cancer cellular immunity (38).

### Activation of CD4^+^ T Cells

**Figure 2:**
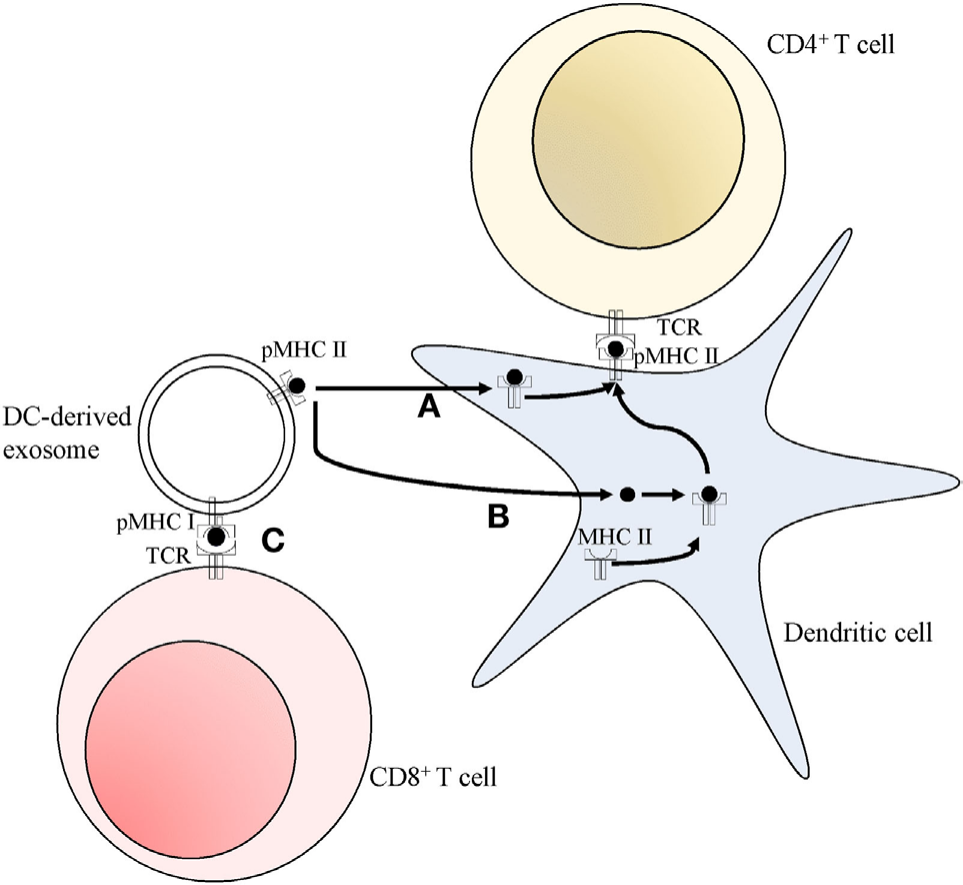
Pathways for T cell activation by dendritic cell (DC)-derived exosomes. **(A)** Peptide MHC class II complexes (pMHC II) can be transferred to DC, and then exposed on the cell surface (cross-dressing). **(B)** Peptides can be used by DC, which load them onto their own endogenous MHC class II molecules, subsequently presenting them at their surface. Both these pathways **(A,B)** allow for the activation of CD4^+^ T cells. **(C)** Peptide MHC class I complexes (pMHC I) can directly interact and activate CD8^+^ T cells in a DC-independent manner.

B cells release exosomes containing significant amounts of functional newly formed MHC class II molecules associated with peptides, along with several accessory molecules, such as B7, ICAM-1, and LFA-3. This enables them to produce powerful *in vitro*, antigen-specific, MHC class II restricted, T helper responses (39). The importance of exosomes in the interactions between T helper cells and B cells was further elucidated by the evidence that the former are powerful stimulators of exosomes synthesis and release from the latter, namely by activation of the CD40 and IL-4 receptors (40–42). B cell-derived exosomes also contain MHC class I molecules, and some components of the B-cell receptor (BCR), such as several tetraspanins, CD19, and immunoglobulin, but not CD21, a normal component of the BCR, present in high quantities on the surface of stimulated B cells (41, 42). It is worth noting that the BCR is an essential piece in the activation of B cells by antigens, leading to the uptake, degradation, and presentation of antigens (43). Exosomes with MHC class II-peptide complexes, which are derived from peptide-pulsed DCs can be taken up by MHC class II-deficient DCs, which use the whole exosomal peptide-MHC complexes to activate T helper cells, a process termed cross-dressing that could contribute to amplify the initial adaptive immune response (Figure 2) (44–46). This process is vastly more efficient when the exosomes are derived from lipopolysaccharide (LPS)-treated mature DCs, in comparison with immature DCs (45). These differences may be accounted for by the significantly smaller amounts of the adhesion molecule ICAM-1 present in immature DCs-derived exosomes (45). Recipient DCs can also use MHC class II-peptide complexes from APC-derived exosomes as a source of antigens which are loaded onto their own MHC class II molecules (Figure 2). This was evidenced by the fact that exosomes carrying IA^b^-IEα_52–68_ complexes can activate CD4^+^ T-cells in wild-type animals (WT), but not in MHC class II^−/−KO^ mice (47) and that Marilyn T-cells, when transferred to MHC class II deficient hosts, are less efficiently activated by H-Y exosomes, when compared with WT animals (48).

The nature of the information transmitted by the exosomes depends also on their cell of origin. For instance, MHC class II-expressing T-84 intestinal epithelial cells release exosomes which strongly activate T cells, through the transfer of peptide antigens to DC-generated MHC class II molecules, rather than transferring the whole MHC class II—peptide complex (49). On the other hand, Di-Hwei Hsu and colleagues showed that DC-derived exosomes transferred pre-assembled peptide-MHC class II complexes to APC, which then go on to activate T cells (50). APC-derived exosomes can also elicit responses by direct interaction between the exosomes and the CD4^+^ T cells, in the case of activated T cells (40). However, the activation of naïve T cells by the exosomes requires an intermediary, such as MHC class II negative DC (40). B cells can also serve as this intermediary if the exosomes are released from LPS-treated mature DC, but not if they originate from immature DC (45).

### Activation of CD8^+^ T Cells

Since all nucleated cells express MHC class I molecules, so do the exosomes secreted by most cells, seemingly giving them the potential to activate CTL (7). However, tumor-derived exosomes can only activate CTL clones after processing by APC expressing the correct MHC haplotype (24, 51). Tumor-derived exosomes contain cancer-related antigens that may permit the initiation of an immune response using DC as intermediaries. Andre and colleagues were able to isolate tumor-derived exosomes from malignant ascites of patients with melanoma, and these were enriched in melanoma-associated antigen (MAGE) recognized by T cells (MART-1). These exosomes, once loaded onto DC, permitted *in vitro* cross-presentation of the antigen, and activation of a clone of CTL, which mounted an efficient *in vitro* anti-tumor cellular response, as measured by the amount of IFN-γ released, and by the promotion of specific tumor cell lysis (24). Furthermore, murine tumor-derived exosomes were shown to contain shared tumor antigens which, once loaded onto human DC, can induce efficient cross-presentation to human CTL leading to *in vivo* cross-protection between different poorly immunogenic mouse tumors (51). These results suggest that tumor exosomes, either collected from tumor cell cultures or directly from malignant effusions, are potential sources of viable antigens for the creation of broad-spectrum immunotherapeutic techniques. Exosomes produced by DC can also activate CD8^+^ T cells indirectly through cross-dressing (50). However, APC-derived exosomes have the additional capacity of directly activating clones of CTL in a DC-independent manner, by cross-presenting exogenous antigens (Figure 2). Saho Utsugi-Kobukai and colleagues demonstrated this by showing that exosomes from ovalbumin peptide-pulsed DCs could stimulate an antigen-specific, MHC class I restricted, T cell hybridoma (52). Results from Charlotte Admyre and colleagues further confirmed this process by showing that exosomes released from monocyte-derived DCs can produce antigen-specific responses on autologous CD8^+^ T cells from human peripheral blood samples (53). They also demonstrated that, much like the case in exosomes activation of CD4^+^ T cells, this process was more efficient when the exosomes came from LPS-treated mature DC rather than immature DC. This difference may be accounted for by the higher concentrations of MHC classes I and II and co-stimulatory molecules on the mature DC-derived exosomes (53).

## Exosomes in Immunosuppression

**Figure 3:**
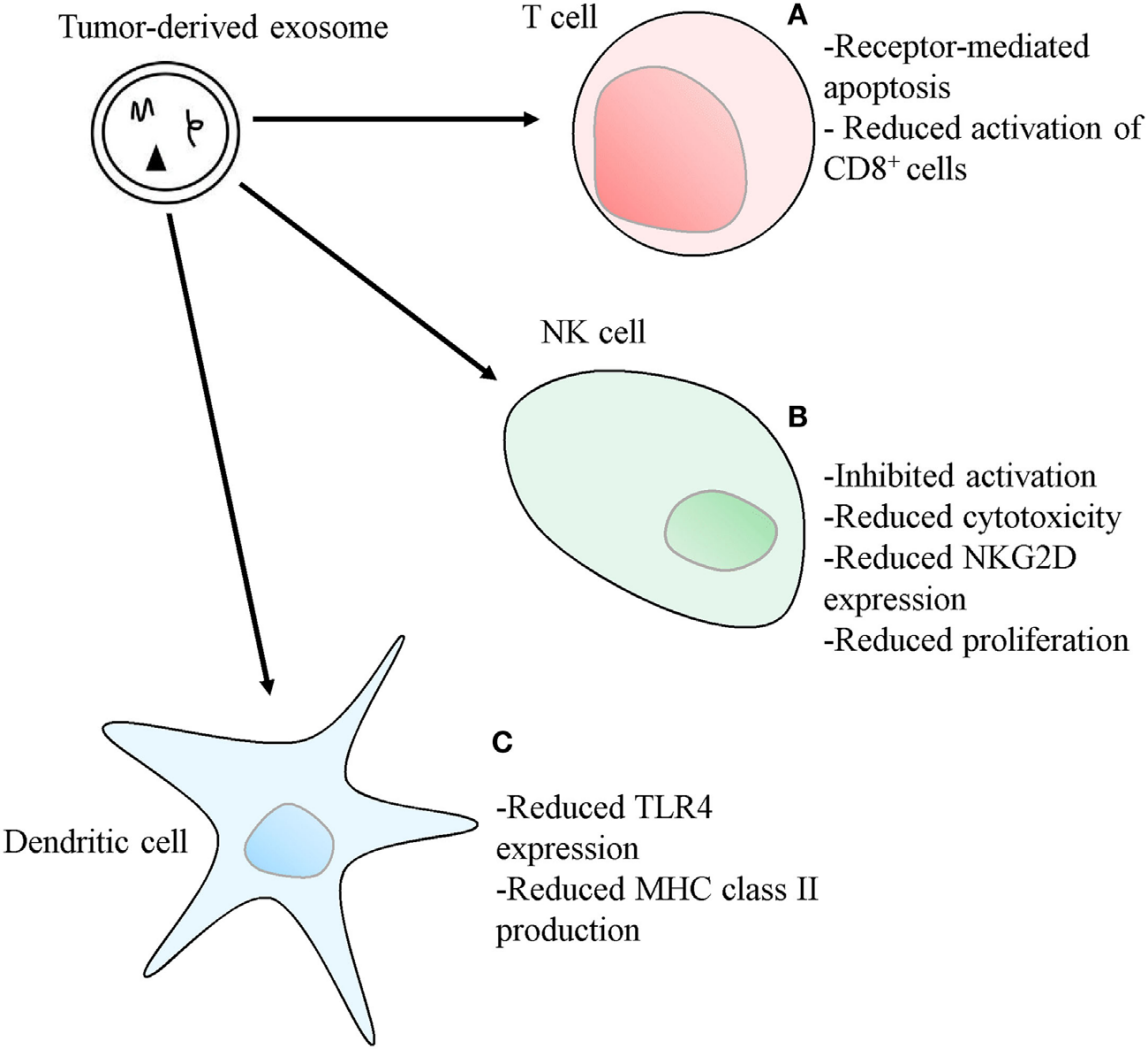
Mechanisms used by tumor-derived exosomes to suppress immune responses. **(A)** Exosomes bearing apoptosis-inducing ligands, such as Fas ligand, TNF-related apoptosis-inducing ligand or, in the case of Th1 cells, galectin 9, can initiate T cell apoptosis. Exosomes also inhibit IL-2-dependent CD8^+^ T cell activation. **(B)** Exosomes bearing TGF-β1 can also disrupt IL-2 signaling to natural killer (NK) cells, thus inhibiting NK cell activation, cytotoxicity, and proliferation. The expression of the NKG2D receptor on NK cells can also be diminished by exosomes carrying NKG2D ligands, thus reducing NK cell ability to recognize malignant cells. **(C)** Exosomes can reduce the expression of toll-like receptor 4 and inhibit the transcription of MHC class II genes in dendritic cells, through the transference of different types of microRNA.

Exosomes are part of the mechanisms cancer cells use to create an immunosuppressive, pro-tumorigenic microenvironment, which allows the disease to progress (54–59). These mechanisms have been observed in numerous cancer types and several different mediators have been identified. A full understanding of these processes may open new avenues for novel therapeutic modalities, such as immune-checkpoint blockade therapies, as viable cancer therapy options. The production and release of exosomes bearing factors capable of inducing apoptosis of the surrounding immune cells, such as Fas ligand (FasL) and galectin 9, is one of the mechanisms used by cancer cells to induce immunosuppression (57, 59, 60). Giovanna Andreola and colleagues showed that melanoma cells accumulate intracellular FasL, namely within MVB, which in this cancer type are characteristically populated by melanin-rich melanosomes (59). The melanoma cells were subsequently shown to release exosomes showing a marked positivity for FasL that were capable of provoking receptor-mediated apoptosis on Fas-sensitive Jurkat T lymphocytes (59). Exosomes induction of apoptosis in activated CD8^+^ T cells was reported by Wieckowski and colleagues (54), and immunosuppression mediated by human colorectal cancer (CRC) cells’ exosomes, bearing both FasL and TNF-related apoptosis-inducing ligand (TRAIL), was demonstrated, also acting through the induction of apoptosis of activated human T lymphocytes (Figure 3) (58). Furthermore, phenotypically similar and pro-apoptotic exosomes were also present in the plasma of CRC patients, demonstrating the *in vivo* release of these vesicles, their potential role in modulating the host’s immune environment, and their possible use as prognostic markers (58). T cell apoptosis induced by FasL-bearing tumor exosomes is significantly inhibited by previously treating the T cells with IRX-2, a cytokine-based biological agent (61). Activated T cells also release exosomes bearing FasL and TRAIL, a process dependent on PKD1/2 (62). These vesicles can induce apoptosis of other activated T cells, in order to prevent autoimmune damage, in a process called activation-induced cell death (AICD) (63).

Pioche-Durieu and colleagues have demonstrated that Epstein–Barr virus (EBV)-infected nasopharyngeal carcinoma (NPC) cells express abundant amounts of galectin 9 (64), a molecule shown to be an agonist of Tim-3 (65). This T_H_1-specific surface molecule mediates the apoptosis of these cells, a mechanism thought to have evolved as another means to prevent prolonged tissue inflammation (Figure 3) (65). Keryer-Bibens and colleagues showed that NPC-derived exosomes bear both galectin 9 and the viral latent membrane protein 1 on their surfaces, also shown to have an intrinsic T-cell inhibitory ability (60). More recently, Klibi and colleagues found circulating galectin 9-containing exosomes in the blood of NPC patients, and reported that these exosomes had the ability to induce apoptosis in EBV-specific CD4^+^ T lymphocytes through the galectin 9/Tim-3 pathway (57). Cancer cells can use exosomes to enact other mechanisms that modulate the immune environment, such as through the interference with cytokine-mediated immune-activation pathways, like those related to IL-1, IL-6, and TGF-β (66–69). Human mesothelioma exosomes were shown to alter the way in which immune cells respond to IL-2 by inhibiting IL-2-driven priming of both cytotoxic NK cells and CD8^+^ T cells, while leaving the IL-2-dependent activation of the immunosuppressive Treg populations unaffected (Figure 3). These effects were mediated by a membrane-associated form of TGF-β1 and show an exosomal “double hit” mechanism tailored to facilitate immune evasion of tumors (66).

Tumor-derived exosomes also promote Treg expansion and increase their immunosuppressive functions. Indeed, Wieckowski and colleagues demonstrated that tumor-derived exosomes, but not DC-derived exosomes, induced a substantial expansion of the CD4^+^CD25^+^FOXP3^+^ Treg population (54). Szajnik and colleagues further clarified this process by showing that tumor-derived exosomes lead to a dose-dependent induction and promotion of Treg proliferation. It was also shown that incubating CD4^+^CD25^neg^ T cells with the same type of exosomes lead to higher percentages of CD4^+^CD25^+^ T cells, indicating that these vesicles mediate this cellular conversion (55). Furthermore, phenotypic changes were also reported, namely an increase in the expression of immunosuppressive cytokines and cytotoxins, such as CTLA-4, FasL, TGF-β1, granzyme B, perforin, and IL-10 in the cells co-cultured with the tumor-derived exosomes, but not in the ones in contact with DC-derived exosomes (55). Immunosuppressive activity changes were also reported in this study, and the Treg cells previously incubated with the tumor-derived exosomes showed increased capacity to induce apoptosis and inhibit proliferation of the responder cells. These effects were also shown to be mediated by both perforin and granzyme B, as the inhibition of these factors impaired the exosomes ability to induce increases in immunosuppressive functions (55). Wada and colleagues showed that exosomes derived from malignant effusions of human patients were able to reduce the decrease in Treg numbers and FOXP3 expression levels in a TGF-β1-dependent manner (70).

Furthermore, tumor-derived exosomes were shown to inhibit NK cell immunity using murine mammary tumor cell lines. When the test animals were submitted to pre-treatment with these exosomes, acceleration in the growth rate of implanted tumors was observed, as well as a decrease in the *in vitro* cytotoxic activity of NK cells incubated with the exosomes (Figure 3) (67). An inhibition of the IL-2-mediated NK cell proliferation, later reported to be mediated by TGF-β1 (66), was also observed (Figure 3) (67).

Murine mammary tumor cells’ exosomes were found to interact with myeloid precursors in the bone marrow, stimulating their release of IL-6 and thus inhibiting their differentiation into DC (68). Human melanoma and CRC exosomes also have the capacity to skew the differentiation of monocytes toward a myeloid line with the ability to suppress T cell function through the release of TGF-β (69). This differentiation modulation is dependent on exosomal Hsp72, on the transcription factor Stat3 (71), and on the adaptor molecule MyD88, involved in toll-like receptor (TLR) family signal transduction (72). Later studies, however, suggest that murine melanoma cells release exosomes which are capable of both promoting maturation of DC and enhancing the T cell-activating capacity of DC (73).

Cancer cells can also modulate the expression of surface receptors on immune cells, such as NKG2D and TLR4 using exosomes. This modulation can be mediated by proteins carried by exosomes, or it can be accomplished through transference of microRNAs to target cells (56, 74–76). The NK cell NKG2D receptor is an important component of cancer immunosurveillance, as cancer cells often aberrantly express NKG2D ligands, which mark them for NK cell-mediated destruction (33). Tumor exosomes bearing NKG2D ligands and TGF-β1 are capable of downregulating the expression of NKG2D on NK cells, and reducing their cytotoxic potential, thus stopping them from recognizing and killing malignant cells (Figure 3) (56, 74). Zhou and colleagues showed that human pancreatic cancer cells’ exosomes are capable of downregulating the expression of the TLR4 in DCs, through the transfer of miR-203, a type of microRNA upregulated in pancreatic adenocarcinoma (Figure 3) (75, 77). It was also reported that the DCs did not have diminished levels of TLR4 mRNA, suggesting that mRNA degradation is not the mechanism behind this receptor modulation, which may occur at the translational level (75). Ding and colleagues, using the same cancer model, reported the release of exosomes containing miR-212-3p which, upon transfer to DC, inhibited regulatory factor X-associated protein (RFXAP), a transcription factor for MHC class II, illustrating yet another DC-suppressing mechanism acting with the help of exosomes (Figure 3) (76). Ying and colleagues demonstrated that human epithelial ovarian cancer cells-derived exosomes were capable of promoting the polarization of macrophages to the tumor-associated immunosuppressive M2 phenotype, and that this transformation was mediated by miR-222-3p, a type of miRNA carried by these exosomes which targets the SOCS3/STAT3 pathway. Furthermore, they demonstrated that these vesicles were also capable of promoting the proliferative and migratory capabilities of ovarian cancer cells (78).

## Exosomes in Immune Activation

Apart from the already mentioned roles exosomes play in antigen presentation, these EVs can also contribute to the promotion of both innate and adaptive immunity through other mechanisms. Macrophages infected with *Mycobacterium avium*, for example, release exosomes containing components from the bacterial cell wall which promote the activation of neighboring uninfected macrophages (79), and numerous other exosomes-mediated processes occur during infections with different types of microorganisms to promote immune responses (80). The role of exosomal immune activation has also been explored in the context of auto-immune disorders, and synovial fibroblasts of rheumatoid arthritis patients were shown to release exosomes containing membrane-bound TNF-α which could inhibit AICD in CD4^+^ T cells (81) and the bronchoalveolar lavage fluid of sarcoidosis patients contained elevated levels of exosomes which could stimulate autologous mononuclear cells (23). Tumor cell-derived exosomes bearing adjuvant molecules, such as heat shock protein (Hsp) 70, can stimulate several different components of both adaptive and innate immune responses, which mount an anti-cancer response (82–85).

Exosomes derived from carcinoembryonic antigen-containing (CEA) tumor cells which were subjected to heat stress, bear Hsp 70 (82). This molecule is a potent adjuvant of immune activation (86, 87), and these vesicles are capable of inducing a more powerful CEA-specific CTL anti-tumor response when compared with exosomes derived from non-heat stressed tumor cells (82). Heat-shocked mouse B lymphoma cells were also capable of promoting anti-tumor immune responses, mostly mediated by CD8^+^ T cells, although CD4^+^ T cells were also necessary, and the exosomes promoted DC maturation (83). Gastpar and colleagues demonstrated that cells from Hsp70/Bag-4-positive human pancreas and colon carcinoma lines release exosomes bearing these same molecules on their surfaces, which stimulate NK cell migration and cytolytic activity (84). Vega and colleagues also showed the role of Hsp70 positive exosomes in the activation of macrophages, as measured by the amount of TNF-α released, which was ≈260-fold higher when compared with recombinant Hsp70 stimulation of the macrophages (85). DC-derived exosomes also bear molecules capable of stimulating immune responses, which represents another argument in favor of the use of cell-free DC-based cancer vaccines, which are explored in more detail in the last section of this review (88–92).

Exosomes derived from mature DC were shown to contain important concentrations of TNF-α. These exosomes were then demonstrated to be internalized by human alveolar epithelial cells, which were so stimulated to release inflammatory mediators, such as IL-8, monocyte chemotactic protein-1, macrophage inflammatory protein 1β (MIP-1β), regulated on activation, normal T cell expressed and secreted (RANTES), and TNF-α. These processes seem to be dependent on the TNF-α cascade (88). Phase I clinical trials involving the administration of DC-derived exosomes in patients with advanced non-small cell lung carcinoma and metastatic melanoma evidenced an activation of NK cells in about half the patients, showing that these exosomes may operate an immune activation of both the innate and adaptive divisions (89–91). This promotion of NK cell proliferation and activation was later shown to be dependent on the expression of membrane-bound functional NKG2D ligands and IL-15Rα, and the inoculation of DC exosomes was also capable of restoring the levels of NKG2D on circulating NK cells of advanced melanoma patients and of inducing tumor regression in mice (92).

## Exosomal Interactions with the Complement System and Opsonins

The complement system, part of the phylogenetically ancient innate immune system, serves as an unspecific recognizer of any invading pathogens. It is capable of triggering the activation of the adaptive immune response, directly catalyzing the destruction of cells by forming the membrane attack complex (MAC), driving systemic reactions through the release of anaphylatoxins, as well as of opsonizing target cells for phagocytosis (93). All vesicular structures in circulation are prone to activating the complement system, leading to their degradation. This process has been described for artificial liposomes designed for therapeutic purposes (94–96) that, despite not being directly antigenic, are capable of activating the complement system in an antibody-independent manner through electrostatic interactions with complement proteins (97). Host cells are naturally protected against the activation of the autologous complement system through the expression of membrane-bound molecules which inhibit it, such as CD59 which prevents the formation of the MAC (98, 99) and CD46 and CD55 which act synergistically to stop the formation and deposition of C3b and C5b (100, 101). APC-derived exosomes, formed in antigen-processing intracellular compartments, are associated with antigenic peptides and should, therefore, be particularly prone to antibody-binding and complement-mediated destruction (97). However, Clayton and colleagues demonstrated the expression of both CD55 and CD59, but not of CD46, on exosomes originated from human monocyte-derived dendritic cells and cells of B lymphocyte origin, which were functional in the *in vitro* inhibition of complement-mediated lysis (97).

An opsonin can be defined as a molecule that binds antigens, marking them for phagocytosis. Numerous molecules can act as opsonins, including antibodies and some members of the complement system. Milk fat globule-EGF-factor 8 (MFG-E8), a protein commonly found on human milk fat globules, was evidenced to act as an opsonin by binding to phosphatidylserine on the surface of dying cells, thus preventing the development of autoimmune diseases by accumulation of apoptotic cells, which can undergo secondary necrosis and release toxic mediators (102, 103). Miksa and colleagues demonstrated that exosomes derived from immature DC, but not mature DC, carried MFG-E8 and were able to restore the effective clearance of apoptotic cells in septic rat models, thus suppressing the pro-inflammatory response and providing protective effects in the context of sepsis (104). This provided a demonstration of the role of exosomes in presenting opsonins (104).

## Cell-Free Cancer Vaccines

Dendritic cells are the most efficient cells at presenting antigens and are the only APC capable of activating naïve T cells and initiating the adaptive immune response (105). Indeed, if we interpret cancer immunosurveillance as a cycle of stepwise events leading to the effective killing of cancer cells by T cells, the cancer-immunity cycle, DC capturing and processing of tumor neoantigens acts as the first step, a process which is dependent on the presence of certain molecular signals, such as pro-inflammatory cytokines, co-stimulatory ligands, molecules released from the dying tumor cells, and gut microbiome products (106). It is then understandable that efficient DC-based cancer vaccines have been long sought after, and some encouraging results using these techniques have already been obtained, such as with the use of Sipuleucel-T Immunotherapy for the treatment of castration-resistant prostate cancer (107). However, the widespread use of DC-based cancer vaccines presents some important limitations (108, 109). The use of DC-derived exosomes (often referred to in the literature as Dexosomes or simply Dex) cancer vaccines has recently emerged as an alternative which may be capable of overcoming some of these difficulties. First, Dex molecular composition is easier to determine, thus facilitating the stricter definition of quality control parameters (46). Dex are also more abundant in peptide-MHC class II complexes allowing for higher yields (46, 109). Dex, when compared with DC, also present a great advantage during long-term storage, because they can safely be frozen for up to 6 months (109). Adding to these advantages, the immunosuppressive tumor microenvironment is often responsible for inhibiting efficient antigen presentation and T cell stimulation by DCs, which should not affect Dex (110, 111). Finally, Dex do not pose most of the risks involved in the administration of viable cells, such as the development of immune dysfunction, or microvascular occlusions (112). The understanding of dexosomes’ viability as immunotherapeutic agents depends on the deep comprehension of their molecular composition. The membranes of Dex contain proteins involved in antigen presentation and T cell activation, such as MHC classes I and II and co-stimulatory molecules, like CD86 (B7-2) (113, 114). Molecules involved in Dex targeting and docking to receptor cells, such as ICAM-1, MFG-E8, and members of the tetraspanin family of proteins, such as CD9 and CD81 are also present in Dex (113–116). Morelli and colleagues described the mechanisms responsible for the targeting of Dex for DC internalization, which was shown to be calcium and temperature-dependent, and to rely on the presence of ligands on the surface of the exosomes, namely MFG-E8, phosphatidylserine, CD11a, CD54, CD9, and CD81, and on the surface of the recipient DC (αv/β3 integrin, CD11a, and CD54) (116). They also provided *in vivo* evidence of bone marrow Dex uptake not only by splenic DC, but also by splenic macrophages and by hepatic Kupffer cells (116). Zitvogel and colleagues provided the proof of concept supporting the *in vivo* efficacy of Dex-based immunotherapy (111). Tumor peptide-pulsed Dex were capable of inducing *in vivo* CTL priming, tumor growth suppression, and tumor remission. Indeed, single intradermal administrations of these exosomes promoted significant tumor growth suppression after a week and, after 60 days, 40–60% of the animals were tumor-free (111). Furthermore, these cell-free immunotherapeutic vaccines were more effective than the direct administration of DC vaccines, which only accomplished a 60th day tumor-free mice fraction of 20%. These differences may be accounted for by the exosomes’ imperviousness to the immunomodulatory effects of the tumor microenvironment, which can impair the ability of DC to present antigens (111). In the past decade, several clinical trials assessing the feasibility, safety, and efficacy of Dex-based cancer vaccines were performed, and the results were generally encouraging. Two 2,005 phase I trials tested this immunotherapy approach, one in advanced non-small cell lung cancer (NSCLC) patients, and the other in metastatic melanoma (MM) patients (89, 90). In both trials the patients received four doses of the vaccine, consisting of autologous Dex loaded with several different MAGE peptides. The vaccine production was shown to be feasible, and the therapy was well tolerated by the patients, with just minor grade 1–2 adverse events (89, 90). Furthermore, some interesting immunological and clinical results were obtained: one-third of the NSCLC patients showed increased systemic immune responses against MAGE, as demonstrated by delayed-type hypersensitivity reactivity, and increased NK cell activity was observed in half of the NSCLC patients analyzed and 8/13 of the MM patients, hinting that Dex exert their effects in both adaptive and innate components of the immune system. Clinically, some of the NSCLC patients also appeared to show prolonged post-immunization disease stability, and one of the MM patients exhibited a minor response with disappearance of one out of three subcutaneous lesions, having remained stable afterward for up to 24 months (89, 90). A more recent phase II clinical trial evaluated the use of IFN-γ-Dex, Dex derived from IFN-γ-stimulated mature DC, as a maintenance immunotherapy after the use of first line chemotherapy in advanced NSCLC patients (117). This study showed the feasibility of production and safety of application of IFN-γ-Dex, with only one out of twenty-six of the patients developing a grade 3 hepatotoxicity. Regarding the clinical outcomes, this trial did not show any objective tumor response, according to the response evaluation criteria in solid tumors. However, it did show that the patients with the longest progression-free survival (PFS) had a significant increase in NK cell function after Dex administration (117).

Tumor-derived exosomes also work as antigen delivery systems, capable of preventing tumor development in a CD4^+^ and CD8^+^ T cell-dependent manner (51). Because of this, cell-free vaccines based on the use of tumor-derived exosomes also emerged as a possibility. This idea, however, presented with a big limitation, since the isolation of tumor exosomes seemed to require the inconvenient *in vitro* culture of the patients’ tumor cells (118). The already mentioned findings of Andre and colleagues explains that malignant effusions of melanoma patients are exosomes-rich, and that these tumor exosomes are capable of transmitting tumor antigens to DC, which then go on to activate tumor-specific CTL capable of mounting an efficient *in vitro* anti-tumor response which offers a solution to the above-mentioned problem (24). Indeed, Dai and colleagues published a phase I clinical trial in which exosomes derived from the ascites of advanced CRC patients were used as immunotherapy (118). These tumor exosomes were administered to the patients in combination with granulocyte macrophage colony-stimulating factor (GM-CSF), a powerful adjuvant which can promote the maturation and function of DC (119). Besides demonstrating the feasibility and safety of this treatment, with only grade 1–2 adverse effects reported, it was also shown that the combination of tumor exosomes with GM-CSF allowed for a more efficient induction of systemic anti-tumor immunity and CTL responses than the administration of the isolated tumor exosomes. Regarding the clinical results, the patients treated with the isolated tumor exosomes showed no therapeutic response, while one patient with stable disease and one patient with a minor response were observed in the group receiving ascites-derived exosomes plus GM-CSF (118).

## Summary and Future Perspectives

Since the first published descriptions of exosomes release from rat reticulocytes (120, 121), the field of exosomes biology grew explosively, and we now know that these EVs, far from being a mere cellular mechanism for waste disposal, play countless roles in intercellular communication. Of particular interest to this review, exosomes were described as key players in the crosstalk between malignant cells and the immune system. Indeed, we know that exosomes can both be promoters of tumor growth and invasion by aiding in the establishment of an immunosuppressive microenvironment and agents at the service of cancer immunosurveillance, by assisting antigen presentation and promoting eradication of tumor cells by CD4^+^ (39) and CD8^+^ (24) T cells and by elements of the innate immune system, such as NK cells (92). The translational applications of exosomes to cancer therapy have been evolving rapidly, with several phase I and II clinical trials evaluating the safety and efficacy of exosomes-based cancer vaccines already published showing promising results which will without a question encourage the development of better models of study in this area with great translational potential (89, 90, 117, 118).

Other therapeutic techniques may benefit from the use of exosomes, such as the delivery of molecules directed against specific cancer targets. Indeed, in collaboration with other peers, we have recently shown that engineered exosomes show better efficacy profiles, when compared with artificial liposomes, in the distribution of interference RNA specific for oncogenic KRAS in pancreatic cancer models. This process is partially dependent on the expression of CD47 on the exosomes, which allowed for their escape from CD11b^+^ monocytes, and consequently increased their half-life. Improved uptake of the exosomes by cancer cells, leading to a more potent anti-cancer activity, and improved survivals were also reported (122).

Given the exosomes’ widespread availability in nearly all body fluids, and the presence of molecules providing insight into the constitution of the cell that released the vesicles, exosomes have also emerged as potentially good biomarkers, allowing for cancer profiling and predicting treatment responses (2). Indeed, research into the value of plasma exosomal content in evaluating responses to chemotherapy and predicting the probability of relapse in acute myeloid leukemia patients has shown promising results (123, 124). These techniques take advantage of the fact that cancer cells release more exosomes than healthy cells (11).

These exciting recent advances in the field of exosomes biology will likely bring profound changes to the lives of cancer patients. They will permit us to use less invasive ways of obtaining the necessary information about the disease, and will open up new therapeutic avenues, more effective, and more individualized, thus minimizing the tremendous side effects most patients still have to currently endure during anti-cancer therapy.

## Author Contributions

SM conceptually designed the manuscript, edited the manuscript, supported, and supervised the writing process. JM advised on the research topic and supervised the writing process. FC supervised the writing process. FB wrote the manuscript and developed the figures.

## Conflict of Interest Statement

SM has ownership interest (patents). No potential conflicts of interest were disclosed by the other authors.
